# Alcoholic Odour Detected in Serous Effusion in the Ventricles of the Brain

**Published:** 1844-05

**Authors:** L. Bradley

**Affiliations:** Elgin, Kane Co. Ill.


*Alcoholic Odour detected in serous effusion in the Ventricles of the Brain.* By L. BRADLEY, M. D., of Elgin, Kane Co. Ill.

*On the 6th of February last,* Doct's. J. & E. Tift, of this village, with myself, were summoned before a Coroner's inquest, holden upon the dead body of Samuel Page, for the purpose of examining the body, and giving testimony in the case.

It appeared, from the evidence before the jury, that the deceased was found about two miles from this village, in his wagon, with his feet hanging over the fore board, his body resting upon a bag of grain, and his head upon the bottom of the wagon. He was totally insensible, as if in deep and heavy sleep; his breathing was stertorous and difficult. He was taken to a neighboring dwelling, where he expired in about ten minutes.

He had, a short time previously, left a "grocery" in this place, where he had been drinking freely; had been on a journey for some days, on his way to Iowa, and had been in the habit of drinking two or three times a day on the road, but was not an habitual drunkard. He left the "grocery" partially intoxicated, without mittens, or any other extra over-clothes, though the weather was somewhat below freezing point. He was a hardy, rugged man, plethoric and robust, with ample chest and a thick short neck.

INSPECTION.

Upon opening the cranium, about six hours after death, dark fluid blood poured rapidly from the sinuses, to the amount of eight or ten oz.—The brain exhibited excessive vascular turgescence; in the corpora striata, a small amount of sanguineous extravasation was detected, and in the lateral ventricles, some serous effusion.

The medical witneses agreed, in expressing their opinion, that the deceased died of apoplexy, caused by an intemperate use of stimulating liquor, and exposure to cold, superadded to a strong predisposition of the system to that disorder.

The verdict of the jury was, "death by apoplexy, caused by intemperance."

The circumstance, and, indeed, the only one, that I have thought rendered this case worthy of particular note, was the fact, that the effused fluid found in the ventricles, yielded strongly, the alcoholic odour; this was so apparent that it was readily recognised by every member of the jury. Thus, we have a fact, corroborating others which have been reported, proving satisfactorily, that in some way, alcohol in substance does find its way to the brain.

I regret to add, that nothing was elicited during the examination, calculated to enlighten us in respect to the mode by which this result is effected.

